# Integration of genome-scale metabolic networks into whole-body PBPK models shows phenotype-specific cases of drug-induced metabolic perturbation

**DOI:** 10.1038/s41540-018-0048-1

**Published:** 2018-02-26

**Authors:** Henrik Cordes, Christoph Thiel, Vanessa Baier, Lars M. Blank, Lars Kuepfer

**Affiliations:** Institute of Applied Microbiology—iAMB, Aachen Biology and Biotechnology—ABBt, RWTH Aachen University, 52074 Aachen, Germany

## Abstract

Drug-induced perturbations of the endogenous metabolic network are a potential root cause of cellular toxicity. A mechanistic understanding of such unwanted side effects during drug therapy is therefore vital for patient safety. The comprehensive assessment of such drug-induced injuries requires the simultaneous consideration of both drug exposure at the whole-body and resulting biochemical responses at the cellular level. We here present a computational multi-scale workflow that combines whole-body physiologically based pharmacokinetic (PBPK) models and organ-specific genome-scale metabolic network (GSMN) models through shared reactions of the xenobiotic metabolism. The applicability of the proposed workflow is illustrated for isoniazid, a first-line antibacterial agent against *Mycobacterium tuberculosis*, which is known to cause idiosyncratic drug-induced liver injuries (DILI). We combined GSMN models of a human liver with N-acetyl transferase 2 (NAT2)-phenotype-specific PBPK models of isoniazid. The combined PBPK-GSMN models quantitatively describe isoniazid pharmacokinetics, as well as intracellular responses, and changes in the exometabolome in a human liver following isoniazid administration. Notably, intracellular and extracellular responses identified with the PBPK-GSMN models are in line with experimental and clinical findings. Moreover, the drug-induced metabolic perturbations are distributed and attenuated in the metabolic network in a phenotype-dependent manner. Our simulation results show that a simultaneous consideration of both drug pharmacokinetics at the whole-body and metabolism at the cellular level is mandatory to explain drug-induced injuries at the patient level. The proposed workflow extends our mechanistic understanding of the biochemistry underlying adverse events and may be used to prevent drug-induced injuries in the future.

## Systems toxicology: linking pharmacokinetics to endogenous metabolism

The genotype of a patient determines the extent of drug-induced metabolic perturbations on the endogenous cellular network of the liver. A team around Lars Kuepfer at Germany’s RWTH Aachen University developed a computational workflow that links drug pharmacokinetics at the whole-body level with a cellular network of the liver. The authors used the competitive cofactor and energy demands in endogenous and drug metabolism to establish a multi-scale model for the antibiotic isoniazid. Their model quantitatively describes how isoniazid pharmacokinetics alter the intracellular liver biochemistry and the utilization of extracellular metabolites in different patient genotypes. The study outlines how a mechanistic understanding of genotype-dependent drug-induced metabolic perturbations may help to explain diverging incidence rates of toxic events in different patient subgroups. This could reduce the occurrence of toxic side effects during drug treatments in the future.

## Introduction

Drug-induced adverse events are a common clinical, and an increasing public health problem.^1^ In many cases, the pathogenesis of such injuries involves the parent drug, as well as its metabolites impairing the cellular homeostasis.^2^ These drug-induced metabolic perturbations can cause oxidative stress, energy shortage, accumulation of triglycerides, or local oxygen depletion. Together, these factors result in cellular dysfunctions and for critical cases in drug-induced toxicities.^3^ Here, drug-induced liver injury (DILI) is one of the most frequent side effect with clinical manifestations in cholestatic, hepatocellular, or mixed forms in an acute or chronic pathological pattern.^4^

Each drug is associated with a characteristic pathway response signature, where the specific pattern of drug-induced injury and its latency is largely determined through drug exposure and hence drug pharmacokinetics.^5^ Drug pharmacokinetics are significantly governed by the underlying ADME processes (ADME: absorption, distribution, metabolism, and excretion). When administered to an organism, drugs are usually recognized as xenobiotic and hence substances, which are potentially harmful to the body. The solubility and clearance of xenobiotic molecules is consequently enforced through sequential activation, modification, and conjugation steps in phase I, II, and III metabolism.^6^ This sequence of biochemical reactions ensures detoxification of xenobiotic molecules, which simultaneously requires the disposition of energy and cofactors. The thereby induced demand of drug metabolism is, however, competitive to the simultaneous requirements of the endogenous metabolism and may in consequence significantly perturb the cellular homeostasis. This mutual competition for cofactors and energy makes the xenobiotic drug metabolism a potential root cause in drug-induced toxicity.^7^ Moreover, a competitive utilization of cellular metabolites not only influences the state of the intracellular metabolic network, it also alters the utilization of metabolites from the exometabolome, which is furthermore reflected by changes in blood metabolite pools of the body.^8^ Therefore, drug-induced metabolic perturbations that impair the intracellular homeostasis and alter exometabolome pools may hold important information about the metabolic state in face of drug exposure and might be used for the identification of biomarker patterns in order to characterize specific cases of drug-induced toxicity.^9^

Drug-induced metabolic perturbations are dependent on the administered drug and its metabolites. Drug metabolism, in turn, is determined by the patient’s physiology and its genetics, as well as the given dose. Taken together, these factors determine the specific manifestation of drug-induced toxicity.^10^ An in-depth understanding of drug-induced biochemical side effects is vital for individual patient safety in terms of dosing, diagnosis, or the design of curative intervention strategies. However, a functional assessment of such side effects inevitably requires the representation of at least two different scales of biological organization: (1) the whole-body level, which determines the pharmacokinetics and thus the exposure of xenobiotics and their metabolites in different tissues and (2) the cellular scale, where the drug-induced (off-target) effects in the metabolic network take place. In clinical practice, organ-specific drug exposure is difficult to assess and is therefore usually approximated by using plasma pharmacokinetics as a surrogate marker. In turn, cellular toxicity induced by a specific drug is typically characterized with in vitro assays. A computational workflow that combines both scales of biological organization would be a viable tool for the understanding of drug side effects and has the potential to substantially improve patient safety.

**Fig. 1:**
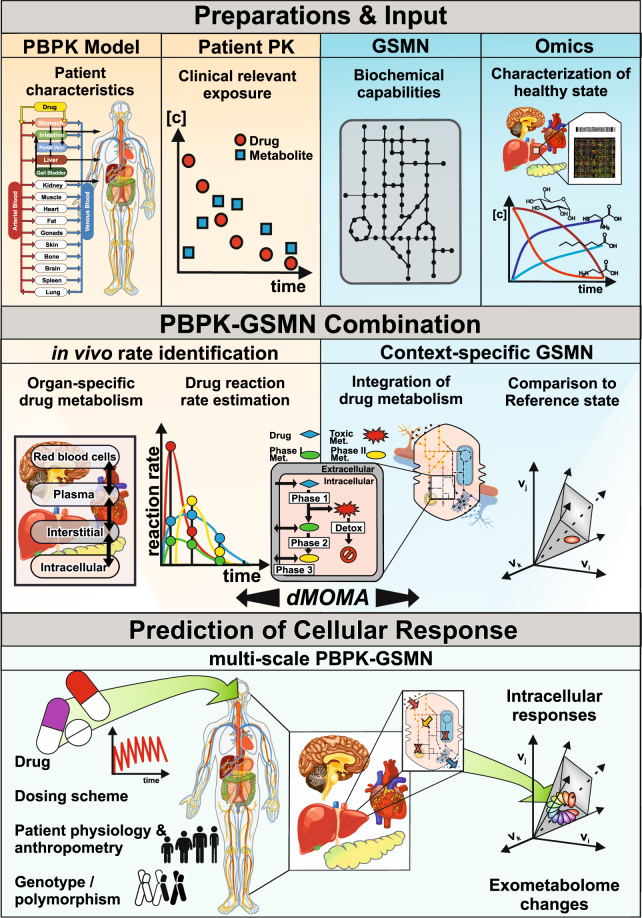
PBPK-GSMN Multiscale modeling workflow. Preparations and input: On the organism level, a comprehensive drug-specific whole-body physiologically based pharmacokinetic (PBPK) model is developed and validated with human pharmacokinetic (PK) data. On the cellular scale, a human genome-scale metabolic network (GSMN) reconstruction is used together with omics data to establish an organ-specific GSMN model and a reference flux distribution. PBPK-GSMN combination: The developed whole-body PBPK model is used to estimate the in vivo organ-specific drug metabolism as time-resolved reaction rates including the absorption, distribution, metabolism, excretion (ADME) processes. The organ-specific GSMN model is extended with the drug-specific xenobiotic metabolism (Table 1). PBPK-derived xenobiotic reaction rates are iteratively used to constrain the xenobiotic reaction rates in the organ-specific GSMN model. A dynamic version of the minimization of metabolic adjustment algorithm (*dMOMA*) is used to calculate altered flux distributions in the drug perturbated organ-specific GSMN. Prediction of cellular responses: The combined multi-scale PBPK-GSMN model can be used to predict organ-specific drug-induced metabolic perturbations, resulting in altered intracellular and extracellular reactions rates. The combined model allows the explicit consideration of specific dosing schemes, patient physiology, and genetic characteristics

**Fig. 2:**
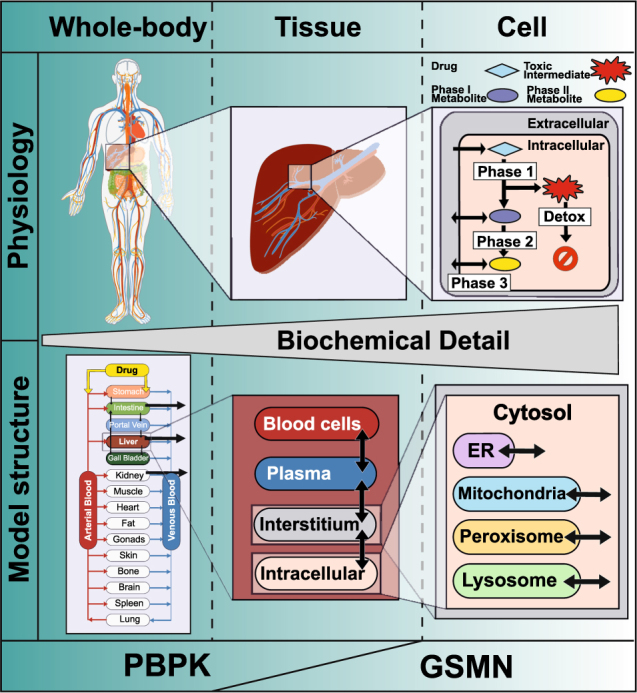
Linking PBPK and GSMN models. Physiologically based pharmacokinetic (PBPK) models cover relevant physiological organs and tissues including the drug-specific ADME processes. Within PBPK models, each tissue is further subdivided into red blood cells, plasma, interstitial, and intracellular compartments. Genome-scale metabolic network (GSMN) models describe the cellular biochemistry in the interstitial (extracellular) and intracellular (cytosol, mitochondria, peroxisome, etc.) space. A combined PBPK-GSMN model connects multiple orders of biological organization ranging from the whole-body down to the cellular level, by integrating the overlapping interstitial and intracellular compartments increasing their level of biochemical detail

In order to investigate drug-induced metabolic perturbations of the cellular metabolism within an in vivo context, we here present a computational workflow that integrates multiple orders of biological organization, ranging from the whole-body, down to the cellular level (Fig. 1). Within this workflow, carefully validated and comprehensive whole-body physiologically based pharmacokinetic (PBPK) models are used to simulate the systemic drug exposure in blood plasma and in tissue compartments of various organs. At the cellular level, genome-scale metabolic network (GSMN) models are used to describe biochemical pathways in the liver and furthermore the impact of drug ADME processes upon the tissue-specific endogenous metabolism and, in consequence, on the exometabolome. Both modeling approaches are connected through the shared xenobiotic reactions of drug and drug metabolites, resulting in a combined PBPK-GSMN model (Fig. 2). Time series of genome-scale flux distributions characterizing the intracellular and extracellular responses in the face of xenobiotic exposure, relative to an unperturbed reference state, are calculated with a dynamic extension of the minimization of metabolic adjustment algorithm^11^ (henceforth referred to as *dMOMA*).

To illustrate the applicability of the proposed workflow we discuss the case of isoniazid, a first-line antibacterial agent against *Mycobacterium tuberculosis* infections causing idiosyncratic DILI.^12^ Polymorphisms in the human N-acetyl transferase 2 (NAT2), the major metabolizing enzyme,^13^ result in acetylator phenotypes with altered pharmacokinetics of isoniazid and various downstream metaolites.^14^ During WHO recommended chemotherapy idiosyncratic DILI events occur frequently in the slow acetylator phenotype.^15^ We combined a GSMN model of a human liver within an extended version of a carefully validated isoniazid PBPK model, which is capable of describing the NAT2-dependent pharmacokinetics of isoniazid and its metabolites in men.^16^ The combined PBPK-GSMN model predicts NAT2-phenotype-specific intracellular responses of isoniazid-induced metabolic perturbations upon the human liver and resulting changes in the exometabolome. The estimated differential responses in cellular pathways and the exometabolome are in line with experimental and clinical findings. The comparison shows, in particular, that phenotypic differences in cellular responses after drug administration can only be explained with combined PBPK-GSMN models, which considers both the cellular biochemistry as well as the highly dynamic xenobiotic metabolism at the whole-body level.

## Results

### The multi-scale PBPK-GSMN modeling workflow

Cellular toxicity is a key manifestation of drug-induced adverse events, which is among others caused by perturbation of the cellular metabolism.^17^ This is due to the fact that energy and cofactors, which are continuously produced in the endogenous metabolism, are both needed to maintain essential cellular functions and to ensure drug detoxification. To mechanistically investigate the competition between xenobiotic and endogenous metabolism, we developed a computational multi-scale workflow that quantifies the dynamic drug-induced perturbations in the intracellular space, and the resulting alterations of metabolite levels in the exometabolome (Fig. 1). The workflow combines organ-specific GSMN models and drug-specific whole-body PBPK models. Both model scales are combined by using the metabolic rates obtained from PBPK simulations to, in turn, constrain the xenobiotic metabolism of organ-specific GSMN models. Genome-scale flux distributions are iteratively calculated with the resulting PBPK-GSMN models that allow a quantitative assessment of cellular responses during drug-induced metabolic perturbations.

At the whole-body level, PBPK models describe the time-resolved ADME processes within the body in large detail. PBPK models are based on physiological knowledge of the organism and on physicochemical information of a drug and its metabolites.^18^ Taken together, this information allows the quantitative estimation of xenobiotic exposure in various organs and their sub-compartments, such as plasma, interstitial, and intracellular space, respectively. Importantly, enzyme-mediated reactions of the xenobiotic metabolism are explicitly represented in PBPK models such that xenobiotic reaction rates are available at each time point.^19^ PBPK models can be assumed to provide accurate quantitative estimates of drug concentrations as well as metabolism and clearance rates in various tissues, if the models were carefully qualified, i.e., the simulated plasma concentration are in agreement with clinical pharmacokinetic data and the overall mass balance is closed.^18^

At the cellular level, GSMN models comprise the set of biochemical transformations in the endogenous cellular network. The basic structure of a GSMN model is given by the cellular reaction stoichiometry. Flux distributions are inherent variables in metabolic networks quantifying the biochemical reaction rates. As such, flux distributions provide a rigorous estimate of the required metabolite and cofactor demands of the current cellular state.

### Model preparation and PBPK-GSMN combination

Both PBPK and GSMN models share the interstitial and intracellular space, which can therefore be used for model coupling. PBPK models quantitatively describe the organ-specific intracellular xenobiotic reaction rates, while GSMN models quantify the consumption of metabolites and cofactors. To combine both model types, the endogenous GSMN model needs to be extended with the xenobiotic reactions present in the PBPK model at their corresponding sub-cellular location (Fig. 2). Since xenobiotic molecules are implicitly balanced by the PBPK model, only the cofactor and metabolite demand of the xenobiotic reactions must be considered (Table 1). Together with organ-specific omics data, the extended metabolic network is tailored into an organ-specific GSMN model for further analyses.

**Table 1:** Cofactor-based integration of xenobiotic reactions into metabolic networks

Xenobiotic reaction	Generic reaction equation	Cellular location
Phase I		
Oxidation	Drug + O_2_ → oxidized drug	Cytosol, mitochondria, peroxisome
Hydrolysis	Drug + H_2_O → hydrolyzed drug	Cytosol, mitochondria, peroxisome
Reduction	Drug + NAD(P)H → reduced drug + NAD(P)^+^	Cytosol, mitochondria, peroxisome
Phase II		
GSH conjugation	Drug + GSH → Drug-GSH	Cytosol
Sulfation	Drug + PAPS → Drug-SO_3_H + PAP	Cytosol
Acetylation	Drug + Ac-CoA → Drug-Ac + CoA	Cytosol
Sugar conjugation	Drug + UDP-SUG → Drug-SUG + ADP	Microsome, endoplasmic reticulum
Methylation	Drug + SAM → Drug-Met + SAH	Mitochondria, nucleus
AA conjugation	Drug + AA → Drug-AA	Mitochondria
Phase III		
Metabolic integration	Drug ( + metabolite) → metabolite	Cytosol, mitochondria, peroxisome, nucleus, lysosome, ER
Transporter	Drug[a] + ATP[a] → Drug[b] + ADP[a] + Pi[a]	Organelle membranes^a^
Sym-/antiporter	Drug[a] + ion[b] → Drug[b] + ion[a]	Organelle membranes^a^

*PAPS* 3′-Phosphoadenosine-5′-phosphosulfate, *PAP* 3′-Phosphoadenosin-5′-phosphat, *GSH* glutathione, *Ac* acetyl group, *SAM* S-Adenosyl-L-methionine, *SAH* S-Adenosyl-L-homocysteine, *AA* an amino acid, *SUG* sugar, *[a], [b]* cellular compartments

^a^Cell surface, mitochondrial, endoplasmic reticulum, golgi apparatus, or vesicles

The quantitative assessment of drug-induced metabolic perturbations in an organ requires the continuous identification of cellular flux distributions, as such characterizing the xenobiotic and endogenous metabolism. To this end, a flux distribution prior to drug administration needs to be established, characterizing an unperturbed reference state. Context-specific extraction algorithms make use of the GSMN model structure to account for metabolic flux activity that is not reflected in gene expression data.^20^ In the used algorithm,^21^ transcriptome levels indicate the likelihood that an enzyme carries a metabolic flux, while metabolite utilization rates represent the cellular physiology and define a reasonable solution space.^22^ The resulting context-specific GSMN model reflects the metabolic capacity of an organ, without further use of a metabolic objective function. The intracellular flux sum of the resulting flux distribution was subsequently minimized, based on the plausible assumption that cells reduce the pathway usage to a most efficient minimum.^23^ This step establishes an organ-specific genome-scale flux distribution that considers in vivo gene expression and physiologically feasible metabolite utilization and was henceforth considered as an estimate of the unperturbed reference state. Integration of the xenobiotic metabolism as well as establishment of the reference flux distribution are mandatory prerequisites to enable the dynamic combination of PBPK and GSMN models, respectively, through their shared reactions of the drug-specific xenobiotic metabolism.

**Fig. 3:**
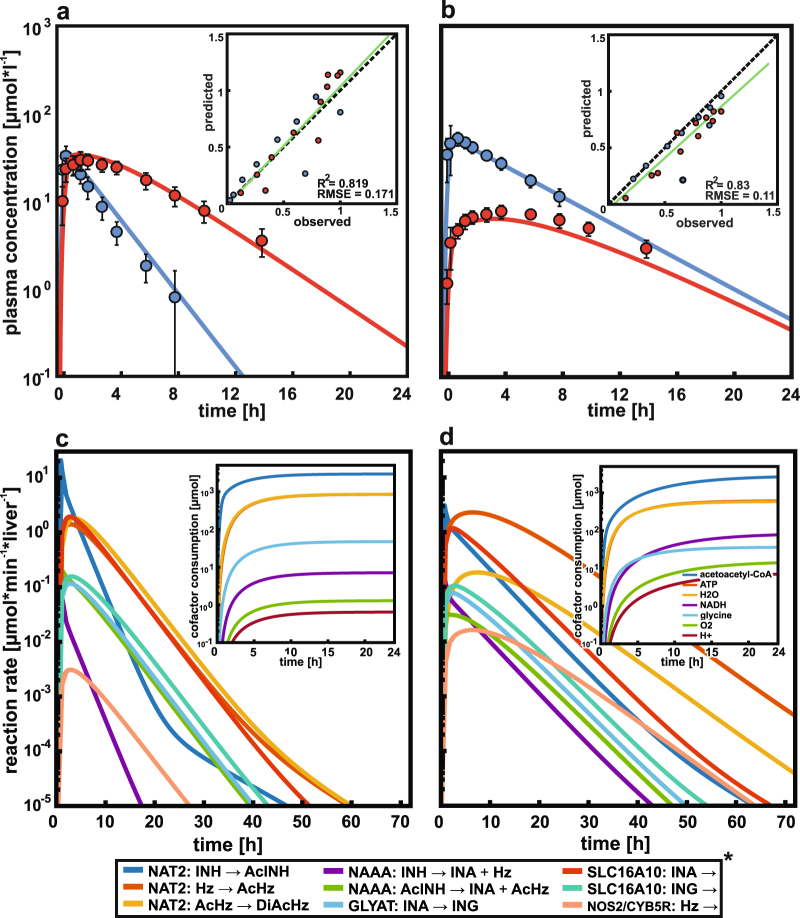
Isoniazid fast and slow acetylator PBPK models and their validation. Physiologically based pharmacokinetic (PBPK) model simulations (lines) and experimental blood plasma profiles (circles)^14^ of isoniazid (blue) and acetylisoniazid (red) in human fast (**a**) and slow (**b**) acetylators after a single oral dose of 300 mg isoniazid. Inserts show the observed^14^ vs. predicted plots. Intracellular reaction rates of the xenobiotic metabolism in the liver after a single oral isoniazid administration in fast (**c**) and slow (**d**) acetylators. Inserts show the cumulative hepatic cofactor consumption, induced by the xenobiotic metabolism of isoniazid. Asterisk indicates intracellular xenobiotic reactions (Supplementary Table 1)

**Fig. 4:**
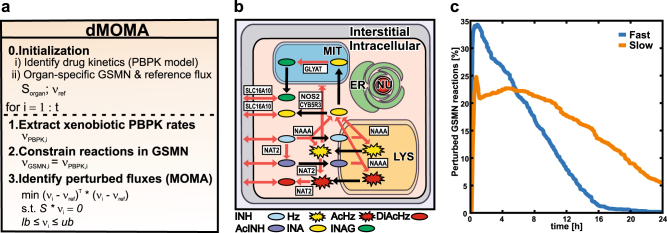
Application of *dMOMA* and GSMN perturbations. Pseudocode of the combination of PBPK and GSMN models with *dMOMA* (**a**), step 0, the initialization, where (i) first the whole-body pharmacokinetics of isoniazid and its metabolites are identified and (ii) second an organ-specific GSMN model (*S*_organ_) with a corresponding reference flux distribution (*v*_ref_) are established. Subsequent steps 1–3 are repeated over the whole simulation time. In step 1, the xenobiotic reaction rate for time point *i* are calculated from the PBPK model (*v*_PBPK*,i*_). Step 2 constrains the xenobiotic reactions in the organ-specific GSMN model (*v*_GSMN*,i*_) with the PBPK-derived reaction rates. In step 3, the MOMA algorithm^11^ is used to identify a new flux distribution (*v*_*i*_) with minimal flux adjustment of the perturbed GSMN model toward the reference flux distribution of the unperturbed state. The xenobiotic metabolism of isoniazid and its metabolites within the liver (**b**), reactions that consume endogenous metabolites (red arrows) are explicitly considered in the hepatic GSMN model (Supplementary Table 1) and are constrained with time-resolved PBPK reaction rates. **c** Fraction of significantly altered reactions in the liver-specific GSMN models in fast (blue) and slow (orange) acetylators predicted by the combined PBPK-GSMN models after a single oral administration of 300 mg isoniazid (see Materials and methods “Cellular responses”)

After the preparatory initialization step, enzymatic reaction and transport rates are iteratively calculated in step 1 for each simulation time point with the PBPK model (Fig. 3). In step 2, these rates are used to update the upper and lower bounds of the corresponding xenobiotic reactions in the organ-specific GSMN model. The updated GSMN model is then used in step 3 to calculate a genome-scale flux distribution that quantifies the impact of drug-induced metabolic perturbations on the cellular metabolism during each time step (Fig. 4a). To this end, *dMOMA* (“Combining PBPK and GSMN models” in Materials and methods) is applied, which estimates the transient metabolic states after a perturbation in a metabolic network, by enforcing principle of biological homeostasis. Thus, transient flux distributions in a perturbed environment remain as close as possible to the unperturbed metabolic reference state. Subsequent analyses of the time resolved flux differences between the reference and the transient metabolic perturbations allow the quantification of drug-induced cellular responses in the endogenous metabolism, as well as in the exometabolome.

### Isoniazid case study

**Fig. 5:**
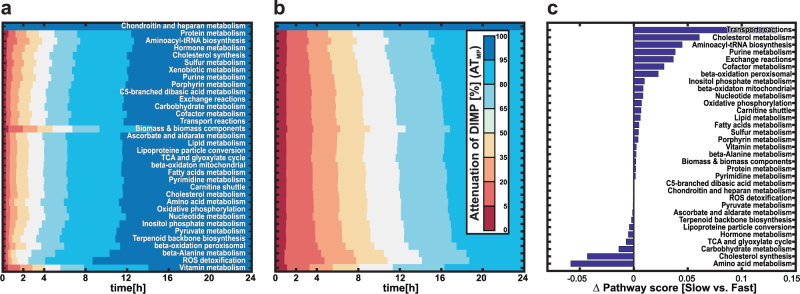
Drug-induced metabolic perturbation (DIMP) upon the biochemical pathways in the liver. DIMP of fast (**a**) and slow (**b**) acetylators shown as the fractional attenuation of clustered biochemical pathway perturbations (*AT*_MP_) (Supplementary Table 3) during a single oral administration of 300 mg isoniazid (see Materials and methods “Cellular responses”). Colors indicate the fractional attenuation of a biochemical pathway perturbation from low (red) to high (blue). **c** Differences in pathway scores over 72 h after a single oral administration of 300 mg isoniazid (see Materials and methods “Cellular responses”). A positive value indicates a higher perturbation in slow and a negative in fast acetylators, respectively

To illustrate the applicability of the proposed workflow we investigated the case of isoniazid, a first-line antibacterial agent against *Mycobacterium tuberculosis* infections.^24^ Polymorphisms of NAT2, the major metabolizing enzyme of isoniazid and its metabolites,^13^ result in acetylator phenotypes with altered pharmacokinetics.^14^ Even during standard chemotherapy, recommended by the WHO, idiosyncratic DILI events occur frequently.^15^ The presented workflow (Fig. 1) was used to combine a previously established NAT2 phenotype-specific PBPK models of isoniazid and its metabolites,^16^ with a GSMN model of the human liver. In a preparatory step, the existing PBPK model of isoniazid was revised and extended with an endogenous hydrazine detoxification reaction to account for experimentally identified pathways.^25,26^ The simulated plasma concentrations of isoniazid and its metabolites are in excellent agreement for fast and slow acetylators (Fig. 3a, b). In accordance with experimental findings,^27^ we found that slow acetylators metabolize less than a quarter of the administered isoniazid dose via the liver, while fast acetylators metabolize nearly two-third of the administered dose (Table 2). Further, about half of the administered dose is excreted uncahnged in slow acetylators, while they metabolize a larger amount of the isoniazid-derived hydrazine moieties (Table 3). The hepatic reactions of the xenobiotic metabolism of isoniazid (Supplementary Table 1) were integrated into a generic model of the human metabolism^28^ at their corresponding subcellular locations (Fig. 4b). Gene expression data of healthy liver biopsies^29^ and metabolite utilization rates^30^ (Supplementary Table 2) were integrated with a model extraction algorithm^21^ to establish a liver-specific GSMN model and a reference flux distribution before drug administration. After these prerequisite steps, *dMOMA* was used to combine the PBPK and GSMN models (Fig. 4a). At peak, about one-third of the reactions in the liver-specific GSMN model were significantly altered about 1 h after isoniazid administration in fast acetylators. In contrast, less than 25% of the hepatic model reactions were affected at peak in slow acetylators (Fig. 4c). The combined PBPK-GSMN models quantitatively show how the isoniazid-induced metabolic perturbations are distributed and attenuated in the liver (Fig. 5a, b). Here, the different xenobiotic reaction kinetics in the liver of fast (Fig. 3c) and slow (Fig. 3d) acetylators are the driving forces of the cellular flux changes in the liver-specific GSMN model.

**Table 2:** Accumulated uptake (+) and secretion (−) of isoniazid and its metabolites in the liver as fraction of administered dose following a single oral administration of 300 mg isoniazid

Compound	Fast (% of dose)	Slow (% of dose)
Isoniazid	70.5	24.1
Acetylisoniazid	−67.7	−22.2
Isonicotinic acid	−1	−1
Isonicotenoyl glycine	−3.2	−2.9
Hydrazine	1.6	−9.5
Acetylhydrazine	32.7	16.2
Diacetylhydrazine	−37.1	−8

**Table 3:** Urinary excretion of isoniazid and its metabolites after a single oral administration of 300 mg isoniazid as fraction of the administered dose

Excreted compound	Fast (% of dose)	Slow (% of dose)
Isoniazid	16.8	53.8
Acetylisoniazid	38.9	12.6
Isonicotinic acid	38.2	28.3
Isonicotenoyl glycine	6.1	5.1
Total isonicotinyl compounds^a^	100	99.8
Total hydrazine liberated^b^	44.3	33.4
Hydrazine	<1	1.3
Acetylhydrazine	<1	2.4
Diacetylhydrazine	40.1	8.4
Total hydrazines^c^	<42.1	12.1
Total hydrazine metabolized^d^	2.2	21.3

^a^Sum of isoniazid, acetylisoniazid, isonicotinic acid, and isonicotinylglycine

^b^Sum of liberated hydrazine moieties from isonicotinic acid and isonicotinylglycine

^c^Sum of excreted hydrazine, diacetylhydrazine, and acetylhydrazine

^d^Difference between total hydrazine liberated and total hydrazine moieties excretion

### Isoniazid-induced cellular responses

A pathway score that reflects the drug-induced metabolic perturbations in clustered biochemical pathways (Supplementary Table 3) was introduced to quantify the cellular effects accompanying an isoniazid treatment (“Cellular Response” in Materials and methods). A pathway score above 1 indicates an aggravation of the drug-induced perturbation in the corresponding pathway compared to the inducing flux in xenobiotic metabolism. A pathway score below 1 suggests a partial perturbation and a pathway score of 0 indicates that the drug-induced perturbation does not affect the pathway. We found that for both, fast and slow acetylators, the administration of isoniazid causes perturbations in a many of metabolic pathways. Simulations with the combined PBPK-GSMN models showed intensified perturbations for extracellular and intracellular transport reactions, the amino acid metabolism, as well as for the synthesis and metabolism of cholesterol in both fast and slow acetylators. In contrast, partial pathway perturbations were found for the remaining pathways, except for the chondroitin and heparan metabolism pathways, which remained unperturbed after isoniazid administration (Supplementary Table 4).

Our simulations show that fast and slow acetylators process the isoniazid-induced metabolic perturbations differentially, although they share the identical xenobiotic reaction stoichiometry. Despite that a greater proportion of hepatic reactions was affected by an isoniazid treatment in fast acetylators, the isoniazid-induced metabolic perturbations were also faster attenuated compared to slow acetylators. In contrast, isoniazid-induced perturbations continued for more than 24 h after drug intact in slow acetylators (Figs. 4c, 5b). However, fast acetylators show higher pathway scores for the amino acid metabolism (0.06), cholesterol synthesis (0.04), and carbohydrate metabolism (0.014). In contrast, slow acetylators had a higher pathway score for intracellular transport reactions (0.13), cholesterol metabolism (0.06), aminoacyl-tRNA-biosynthesis (0.045), exchange reactions (0.04), purine metabolism (0.04), and cofactor metabolism (0.03) (Fig. 5c).

### Exometabolome changes allow prediction of genotype-specific biomarker signature

**Fig. 6:**
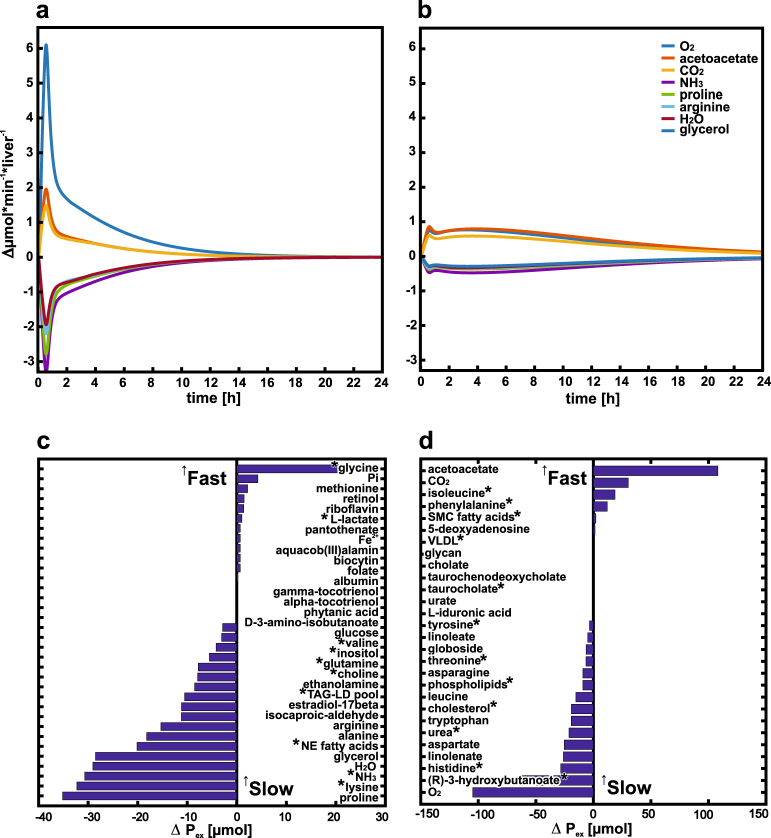
Isoniazid-induced alterations perturbations in the hepatic exometabolome. Predicted changes in hepatic metabolite utilization rates after an oral administration of 300 mg isoniazid for exemplary exometabolome compounds in fast (**a**) and slow (**b**) acetylators. Isoniazid-induced perturbations in the increased (**c**) and decreased (**d**) hepatic exometabolome pools (*P*_ex_, see “Cellular responses” in Materials and methods) between slow and fast acetylators over 72 h after an oral administration of 300 mg isoniazid. A positive value indicates higher metabolite amounts in the exometabolome in slow, negative values in fast acetylators, respectively. Asterisk: Known from literature to be altered after isoniazid administration (Supplementary Table 5)

In addition to direct changes in the intracellular metabolic network, drug administration influences the cellular physiology described by the exchange of metabolites between the intracellular and extracellular environment. The administration of isoniazid caused NAT2 phenotype-specific response patterns of hepatic metabolite utilization rates (Fig. 6a, b). The integration of altered metabolite utilization rates (“Cellular response” in Materials and methods) lead to accumulated changes of more than 50 µmol for many metabolites in the exometabolome (Supplementary Table 5, Supplementary Fig. 4). Notably, metabolite utilization from exometabolome pools varied between fast and slow acetylators, with glycine, proline, lysine, ammonia, and glycerol being most affected ones in increased (Fig. 6c), and acetoacetate, oxygen, and (R)-3-hydroxybutatnoate in decreased exometabolome pools (Fig. 6d), respectively.

## Discussion

In this work, a generic computational multi-scale workflow that combines whole-body PBPK and organ-specific GSMN models for the mechanistic assessment of drug-induced metabolic perturbations is presented (Fig. 1). The resulting multi-scale PBPK-GSMN models allow the quantification of organ-specific endogenous cellular responses and changes in exometabolome pools. Notably, the workflow is fundamentally based on the MOMA algorithm,^11^ but extends it to the dynamic situation of drug-induced flux alterations of the endogenous metabolism relative to an unperturbed reference state. Deviations from the reference state in the combined PBPK-GSMN models are driven by drug pharmacokinetics and their underlaying ADME processes. The combined PBPK-GSMN models therefore enable the tracking of the biochemical responses in specific organs in face of the highly dynamic changes in cofactor demands induced by drug pharmacokinetics.

### Previous work with PBPK-GSMN models

We have previously introduced a generic approach for the coupling of dynamic PBPK and GSMN models.^31^ While acetaminophen intoxication was already analyzed in this initial study, the drug-induced perturbation of the cellular metabolism was, however, rather qualitatively addressed through the impaired capability of a metabolic network to fulfill a set of predefined metabolic tasks. The previously established concept of combining PBPK and GSMN models has been further applied for modeling the interstitial uptake of levodopa,^32^ cortisol signaling,^33^ diabetes,^34^ and the impact of phenytoin on estradiol metabolism.^35^ In complementary approaches, stoichiometric models have been considered within a whole-body context by using static multi-tissue GSMN models to investigate the endogenous metabolic interplay in diabetes^36^ and for the analysis of the impact of different diets on the human metabolism and xenobiotic reaction stoichiometry.^37^ We here extend the original concept significantly, by combining dynamic PBPK models with organ-specific GSMN models through shared reactions of the xenobiotic metabolism. The presented approach allows in particular an accurate description of the highly dynamic interplay of xenobiotic and endogenous metabolism as exemplified here for idiosyncratic DILI events caused by isoniazid pharmacogenomics. Dynamic genome-scale flux distributions allow a dense tracking of transient cellular responses at the molecular level in face of drug exposure.

### The rational of *dMOMA* in combined PBPK-GSMN models

Environmental stresses such as drug exposure are known to alter intracellular flux distributions, as well as exometabolome pools.^38^ In these situations, maintaining the cellular homeostasis against extracellular perturbations is essential to ensure cell viability.^39^ A sustained cellular homeostasis enables to compensate for sudden changes in metabolite availability and demands. Importantly, homeostasis thus enables to distribute and attenuate metabolic perturbations to minimize the impact on single reactions on the whole metabolic network.

Here, the application of *dMOMA* is of particular relevance, since a universal metabolic objective function that could be otherwise considered for constraint-based simulations of healthy tissue or organs is unknown to date. Likewise, metabolic tasks cannot be considered as a true representation of genome-scale flux distributions for a healthy metabolism, since they mostly focus on single reactions or pathways.^40^ Further, these metabolic objectives have not been systematically examined for human cells or tissues.^41^ Originally, MOMA was developed to evaluate metabolic flux distributions in a suboptimal state, following a genetic perturbation.^11^ In the past MOMA was used to show that cells are evolved to achieve their various metabolic tasks with efficient use of energy,^42^ to predict genome-scale flux distributions reflecting near-zero growth of healthy human cells^43^, and to appropriately predict transient metabolic states after genetic perturbations.^44^ Metabolic reaction rates change far more quickly than gene expression and translation. Thus, sudden changes in metabolite demands are compensated by minimal changes of intracellular fluxes, maintaining the previous metabolic steady-state without rigorously changing gene expression.^45^ MOMA enforces this rational of metabolic homeostasis that enables a cell to remain as close as possible to its evolved optimal metabolic state as cellular objective.

For the integration of PBPK and GSMN models, *dMOMA* was used as cellular objective, given a set of metabolic reaction rates calculated by a PBPK model for each time point (“Combining PBPK and GSMN models” in Materials and methods). By following the pharmacokinetic profile, the presented approach results in a time series of transient flux distributions reflecting the drug-induced metabolic perturbations as flux changes in the GSMN evoked by the administration of a xenobiotic compound (Supplementary Fig. 1).

### Isoniazid use case

We illustrated the applicability of the presented workflow by exemplarily investigating the drug-induced metabolic perturbation of isoniazid in the human liver. A comprehensive PBPK model of isoniazid and its metabolites was established and carefully validated before.^16^ This model could in particular describe the impact of a genetic polymorphism in NAT2 on isoniazid pharmacokinetics.^13^ The combination of isoniazid whole-body PBPK models with liver-specific GSMN models showed that isoniazid exposure and the subsequent detoxification processes perturb the endogenous hepatic metabolism of fast and slow acetylators differentially (Figs. 4c, 5, 6). Here, the intracellular and extracellular transport, amino acid, cholesterol, carbohydrate, and lipid metabolism were most affected by isoniazid in both NAT2 acetylator types which is in agreement with previous (pre-)clinical results.^46,47^ Although fast and slow acetylators have the identical xenobiotic reaction stoichiometry, the NAT2-dependent isoniazid pharmacokinetics lead to altered drug-induced metabolic perturbations of cellular pathways, and in consequence, of the exometabolome pools. This clearly indicates the importance to account for both the intracellular network responses as well as the dynamic drug pharmacokinetics when assessing manifestations of drug-induced injuries. Combined PBPK-GSMN modeling further revealed that biochemical pathway perturbations are sustained in slow, compared to fast acetylators (Fig. 5a, b). This was in agreement with a simulation of seven consecutive administrations of 300 mg isoniazid in fast and slow acetylators, which showed a continuous perturbation of more than 5% of the GSMN model reactions in slow acetylators (Supplementary Fig. 3). Since isoniazid is usually administered in a once daily regimen,^24^ a continuous perturbation of endogenous biochemical reactions and pathways occur in slow acetylators. This could explain the increased incidences of idiosyncratic DILI events in this population subgroup after several weeks or months of therapy.^48^

Besides the perturbations of cellular pathways in response to isoniazid administration, the hepatic physiology characterized by its metabolite utilization was found to be significantly altered in fast and slow acetylators. Here, the highest alterations were found for oxygen, ammonia, amino acids, fatty acids and their metabolites, cholesterol, choline, and inositol. The signature of the isoniazid-induced metabolic perturbations in the exometabolome is in broad agreement with (pre-)clinical studies, where the same metabolites were found altered in the plasma of human patients^49,50^ and animal models^51^ (Fig. 6).

In this regard, targeted in vitro strategies that mimic organ-specific in vivo drug exposure profiles could provide more accurate data of cellular response in the future.^52^ Vice versa, the integration of different omics data types of such studies would allow the validation and re-calibration of the endogenous metabolic network, for example, after multiple drug administration.

In conclusion, we showed that the proposed computational multi-scale workflow based on PBPK-GSMN models allows the prediction of drug-induced metabolic perturbations at cellular pathway level, which may ultimately lead to fatal DILI events. Furthermore, the quantification of changes in the exometabolome pools following drug administration could be used for the identification of potential drug and organs-specific biomarker signatures. We here exemplarily investigated isoniazid-induced metabolic perturbations in the human liver and found that an accurate representation of the drug metabolism as well as the resulting pharmacokinetics are crucial, given the highly dynamic conversion of parent drug and drug metabolites. Notably, the presented workflow is generic and therefore not limited to isoniazid or idiosyncratic DILI cases, but can rather be applied to any combination of drug and off-target tissue to address for example cases of nephrotoxicity or cardiotoxicity. This is of particular relevance, since modern drug therapies increasingly aim for personalized treatment regimens to optimize risk-benefit ratios for individual patients.^53^ A mechanistic understanding of the complex interplay of a patient’s physiology and genetics is for example mandatory to account for inter-individual variability in patient cohorts.^54^ In this regard, PBPK-GSMN modeling can be used to simulate drug-induced metabolic perturbations in patients and to predict individual functional endpoints for efficacy, safety, and toxicity. PBPK-GSMN modeling may therefore be a valuable tool for drug research and development in the future to establish personalized dosing regimens, identify biomarker signatures, or design metabolic intervention strategies leading to optimal risk-benefit ratios in patient care.

## Materials and methods

### Whole-body PBPK modeling

The human PBPK models of isoniazid were built with the PBPK modeling software PK-Sim^®^ (Version 7.1.0; Bayer AG, 2017). Model parameter identification was performed in MATLAB (Version 8.5.0.197613; The MathWorks Inc., Natick, MA) and MoBi^®^. The latest versions of PK-Sim^®^ and MoBi^®^ are freely available under the GPLv2 License (https://github.com/Open-Systems-Pharmacology). Physicochemical compound properties (lipophilicity, water solubility, molecular weight, and pKa values) of all modeled compounds were estimated with MarvinSketch (Version 15.11.30.0; ChemAxon Kft., Budapest, Hungary) and used to parameterize the basic distribution model in PK-Sim^®^. The PBPK model of isoniazid and its metabolites was used to simulate the pharmacokinetics of a single oral administration of 300 mg isoniazid for both human fast and slow acetylators. Here, a total simulation time of 72 h was used to ensure complete metabolism and wash-out from the body. The workflow of PBPK model development, including parent drugs, metabolites, and model validation, is described in detail elsewhere.^18^ The detailed development of the used PBPK models of isoniazid and its metabolites as well as their validation was described earlier.^16^ Literature data published as article figures used to parametrize and validate the PBPK model were digitalized and extracted with the WebPlotDigitizer (https://automeris.io/WebPlotDigitizer). The previously described isoniazid PBPK model was updated for a hepatic hydrazine clearance reaction (NOS2; UniProtKB: P35228) and re-parameterized. The used PBPK models of isoniazid and its metabolites are available at: https://github.com/HenrikCordes/isoniazid-PBPK-model.

### Stoichiometric network modeling

Reactions of the xenobiotic metabolism were retrieved from literature^55^ and databases,^13,56^ and incorporated into a genome-scale reconstruction of a generic human cell.^28^ All reactions of the xenobiotic metabolism were integrated into the metabolic network at their corresponding subcellular locations.^57^ Since the dynamic drug and drug metabolite profiles were implicitly balanced in the PBPK models, only the cofactor stoichiometry was considered within the GSMN models (Supplementary Table 1).

Stoichiometric modeling was performed within using the COBRA toolbox^58^ and the gurobi solver (Gurobi Inc.). Gene expression data of healthy liver biopsies^29^ and hepatic metabolite utilization data^30^ was used together with the integrated metabolic analysis tool (iMAT)^21^ to prune a generic human cell^28^ into a context-specific GSMN model of a healthy human liver. The unperturbed liver biochemistry was simulated in a fasted state, where the uptake of gluconeogenic substrates, non-esterified fatty acids, and amino acids, as well as gases and minerals (oxygen, phosphate, etc.), was allowed. In turn, the model could secrete glucose, urea, VLDL, ketone bodies, and albumin, respectively. Further, utilization rates of key metabolites were set as lower and upper bounds (Supplementary Table 2).^30^

### Reference state identification

Differential gene expression data of healthy liver biopsies (GSE74000)^29^ were averaged and filtered for genes in the generic human cell. The data was then normalized to the maximal expression value, and translated to reaction-based expression scores.^59^ The 75th percentile of the cumulative non-zero intensity distribution was used as threshold for the active and the 25th percentile for the inactive set in the iMAT algorithm. Further, the minimal flux threshold *ε* = 10^−5^ in µmol liver^−1^ min^−1^ was used for the iMAT algorithm and a minimal flux (10^−4^ in µmol liver^−1^ min^−1^) was constrained through the biomass reaction. Appling iMAT resulted in a liver-specific GSMN model together with a consistent flux distribution. Subsequently, intracellular fluxes were minimized, while the previously identified flux directionalities and metabolite exchange rates were maintained. The resulting flux distribution was used as reference flux distribution (*v*_ref_) of the healthy unperturbed state of the liver, before drug administration.

### Combining PBPK and GSMN models

The liver-specific GSMN model was combined with the dynamic whole-body PBPK models by stepwise discretization into integration time steps.^60^ For each integration step, the xenobiotic reaction and transport rates in the intracellular PBPK model compartment of the liver were extracted and used as constraints for the lower and upper reaction bounds in the liver-specific GSMN model. Notably, all other hepatic metabolite utilization and intracellular reaction rates were left unconstrained during the coupling. A dynamic version of the minimization of metabolic adjustment (*dMOMA*) (Fig. 4a) was then used for model optimization and applied for each integration step, by using the previously identified flux distribution before drug administration as a reference (wild-type flux solution). The MOMA algorithm was originally developed to evaluate metabolic flux distributions in a suboptimal state, following a genetic perturbation.^11^ It was shown that MOMA appropriately predicts transient metabolic states after genetic perturbations.^44^ In the context of drug-induced metabolic perturbations, MOMA is used to identify the transient flux distributions after drug administration that are as close as possible to the reference flux distribution. MOMA enforces the reference flux distribution in the unperturbed state as objective function, while satisfying the xenobiotic reaction rate constraints that lead to the experimentally observed pharmacokinetic of isoniazid and its metabolites in human in blood plasma. Iteratively applied on the pharmacokinetic reaction profile, MOMA allows to evaluate the impact of xenobiotic reaction activity on cellular flux distributions. *dMOMA* was applied over the whole PBPK model simulation to calculate a time series of flux distributions with respect to the original reference state. A step size of 1 min was used for the integration (step sizes of 1 and 10 min were tested both resulting in the same dynamic flux profiles; Supplementary Fig. 2). For each time step, the following optimization problem was solved:

1 $$\begin{array}{l}{\mathrm {min}}\left( {v_i - v_{\mathrm {ref}}} \right)^T\left( {v_i - v_{\mathrm {ref}}} \right)\\ {\mathrm{s}}{\mathrm{.t}}{\mathrm{.}}\quad \quad S^\ast v_i = 0,\end{array}$$

2 $$lb \le v_i \le ub,$$

3 $$v_{\mathrm {PBPK}} = v_{\mathrm {PBPK},i},$$

where *S* is the *m *× *r* stoichiometric matrix of the organ-specific GSMN model with *m* metabolites and *r* reactions, *v*_PBPK,*i*_ is the set of xenobiotic reaction rates in the intracellular compartment of the PBPK model for a time point, *v*_PBPK_ is the corresponding set of xenobiotic reactions in the GSMN, and *v*_ref_ is the previously identified reference flux distribution. The coupling of PBPK and GSMN models resulted in a series of flux distributions, forming the flux matrix (*v*_*i*__*t*_). Here, each row *i* contains a reaction trajectory with the time resolved biochemical reaction rates over the whole simulation time *t*.

### Cellular responses

Differential fluxes (Δ*v*_*it*_) were calculated for each flux and every time point in the flux matrix with respect to the corresponding reference flux distribution (*v*_ref_).

4 $$\Delta v_{it} = v_{it} - v_{\mathrm {ref}}.$$

Here, a positive difference indicates an increased flux in response to the drug-induced metabolic perturbations of the xenobiotic metabolism, while a negative difference indicates a reduced flux, with respect to the corresponding reference flux.

Integrating the differential fluxes (Δ*v*_*it*_ in µmol liver^−1^ min^−1^) over the whole simulation time *t* results in the accumulated perturbation of a metabolic flux after drug administration (*P*_*rxn*_ in µmol liver^−1^). In case of the xenobiotic metabolism (PBPK), the integrated perturbation (*P*_PBPK_) is an estimate for the total impact of a drug perturbation on the endogenous metabolism, evoked by all organ-specific drug ADME processes. Integrated differential metabolite utilization reactions (exchange reactions, *ex*) (*P*_ex_) estimate the altered amount of an exchanged metabolite (in µmol) in the exometabolome pool of an organ.

5 $$P_{rxn} = \mathop {\int }\limits_0^t \left| {\Delta v_{i}} \right|dt,\;P_{\mathrm {ex}} = \mathop {\int }\limits_0^t \left| {\Delta v_{\mathrm {ex}}} \right|dt,\;P_{\mathrm {PBKP}} = \mathop {\int }\limits_0^t \left| {\Delta v_{\mathrm {PBPK}}} \right|dt.$$

Similarly, the drug-induced metabolic perturbations (*P*_*MP*_) of a metabolic pathway (MP) consists of the accumulated perturbations of its perturbed reactions *k*:

6 $$P_{\mathrm {MP}} = \mathop {\sum }\limits_{rxn = 1}^k P_{rxn} \in \mathrm {MP}.$$

The attenuation of a drug-induced perturbation in a biochemical pathway (*AT*_MP_) was estimated by normalizing the time series of pathway perturbations at each time point *j* (*P*_MP*,j*_) with respect to the cumulative pathway perturbation of the whole simulation (*P*_MP_):

7 $$AT_{\mathrm {MP}} = \mathop {\sum }\limits_{j = 0}^t P_{\mathrm {MP},j}/P_{\mathrm {MP}}.$$

Cellular responses caused by a drug-induced metabolic perturbation were estimated with a pathway score. Biochemical pathways of the organ-specific GSMN models were clustered for metabolic similarity (Supplementary Table 3) and the reaction-based perturbations (*P*_*rxn*_), associated with a clustered biochemical pathway were filtered for significantly altered fluxes, integrated, and normalized to the drug-induced metabolic perturbation of the xenobiotic metabolism (*P*_PBPK_). Notably, a flux was considered significantly altered, if *P*_*rxn*_ was greater than the integrated minimal flux threshold *ε* (*P*_*rxn*_ > ε*t).

8 $$\mathrm {PS} = P_{\mathrm {MP}}/P_{\mathrm {PBPK}}.$$

As such, a pathway score is an estimate for the response of a cellular pathway relative to the metabolic perturbations induced by the xenobiotic metabolism after drug administration. A pathway score above 1 indicates an aggravation of a drug-induced metabolic perturbation, a pathway score between 0 and 1 indicates a partial pathway perturbation, and a pathway score of 0 independence.

### Data availability

Supplementary Information includes a detailed description of the xenobiotic metabolism of isoniazid, definitions, tables, and figures. All data supporting the findings of this study are available within the paper and Supplementary Information. Supplementary Information is freely available at *NPJ Systems Biology and Applications* website. The used PBPK models of isoniazid and its metabolites are available at: https://github.com/HenrikCordes/isoniazid-PBPK-model.

## Electronic supplementary material

Supplementary information

Supplementary Table 1—Xenobiotic reactions of isoniazid in the GSMN

Supplementary Table 2—Metabolite utilization rates

Supplementary Table 3—Exometabolome changes

Supplementary Table 4—Biochemical pathways

Supplementary Table 5—Pathway Scores

## Electronic supplementary material

**Supplementary information** accompanies the paper on the *npj Systems Biology and Applications* website (10.1038/s41540-018-0048-1).
