# Supplementary material for: Integration of genome-scale metabolic networks into whole-body PBPK models shows phenotype-specific cases of drug-induced metabolic perturbation
Source: NPJ Syst Biol Appl. 2018 Feb 26;4:10. doi: 10.1038/s41540-018-0048-1 (PMC5827733; doi:10.1038/s41540-018-0048-1)
Supplement: Supplementary file 1 — Supplementary information [file 41540_2018_48_MOESM1_ESM.docx]

# Supplementary Information

**Integration of genome-scale metabolic networks into whole-body PBPK models shows phenotype-specific cases of drug-induced metabolic perturbation**

**Authors:** Henrik Cordes^a^, Christoph Thiel^a^, Vanessa Baier^a^, Lars M. Blank^a^, Lars Kuepfer^a^

**Affiliations:** ^a^Institute of Applied Microbiology - iAMB, Aachen Biology and Biotechnology – ABBt, RWTH Aachen University, Germany

## Isoniazid metabolism in whole-body PBPK model

The used isoniazid (INH) PBPK model^1^ comprises the parent drug INH and its metabolites acetylisoniazid (AcINH), isonicotinic acid (INA), isonicotinylglycine (INAG), acetylhydrazine (AcHz), diacetylhydrazine (DiAcHz), and hydrazine (Hz). Isoniazid is acetylated by N-acetyltransferase 2 (NAT2) to form AcINH. The N-acylethanolamine acid amidase (NAAA) was defined to catalyze the hydrolyzation of AcINH into INA and AcHz. Isonicotinic acid is conjugated with glycine by an unknown transferase to form INAG. Based on chemical and structural similarity of INAG and hippuric acid, glycine-N-acyltransferase (GLYAT) was assumed to catalyze this reaction.^2,3^ Due to its broad substrate spectrum,^4^ the basolateral T-type amino acid transporter (SLC16A10) was introduced for active INA and INAG transport. Besides the acetylation pathway, INH can be directly converted into INA and Hz. The NAAA was assumed to catalyze this reaction, since the molecular site of reaction is the same as in the hydrolysis of AcINH to INA and AcHz. Hydrazine is acetylated by NAT2 to AcHz and further acetylated by NAT2 to DiAcHz.^5^ Besides acetylation and subsequent exertion, Hz can be metabolized by nitric oxide synthase 2 (NOS2).^6^ All active metabolization reactions NAT2_INH_, NAT2_Hz_, NAT2_AcHz_, NAAA_INH_, NAAA_AcINH_, GLYAT_INA_, and NOS2_Hz_ and active transport reactions processes SLC16A10_INA_, and SLC16A10_INAG_ were implemented with Michaelis-Menten kinetics. In addition to the active metabolization reactions, renal excretion reactions for INH, AcINH, INA, INAG, Hz, AcHz, and DiAcHz were considered as glomerular filtration process. The INH conjugation reactions with α-ketoglutarate or pyruvate were not considered as enzymatic processes and lumped together into the active tubular secretion process, since no pharmacokinetic data for those metabolites was available.

The complex metabolism of INH and its metabolites was implemented in PK-Sim® & MoBi® modeling software (Version 7.1.0; Bayer AG, 2017; available at: <https://github.com/Open-Systems-Pharmacology>). Physicochemical compound properties such as lipophilicity, water solubility, molecular weight, and pK_a_ values were calculated with MarvinSketch (Version 15.11.30.0; ChemAxon Kft., Budapest, Hungary) and used to parameterize the basic distribution model in PK-Sim®. Tissue-specific enzyme availability for all enzymatic processes was quantified by using gene expression levels as a surrogate for protein abundances.^7^

## Isoniazid metabolism in metabolic network models

The cofactor based reactions of the INH pharmacokinetics in the PBPK model were added to the generic model of the human metabolism (Supplementary Table 1). While it is known that hydrazine can be oxidized by NOS2,^6^ experimental animal studies also showed that hydrazine is further metabolized to nitrogen^8^ and ammonia.^9^ Therefore it was assumed, that INH derived Hz (N_2_H_4_) moieties are metabolized by NOS2 forming, besides ammonia (NH_3_), a hydroxylamine molecule (NH_2_OH) under the consumption of NADPH (CYB5R3_Hz_; EC: 1.14.13.39; EntrezID: 4843):

$$N_{2}H_{4}+NADPH+H_{2}O\to{NH}_{3}+{NH}_{2}OH+{NAPD}^{+}$$

To mimic an *in vivo* like situation, both the nitrogen and ammonia biochemical metabolization pathways were integrated in the metabolic network. Here, an equal flux into both pathways via the NOS2 generated hydroxylamine was assumed (1:1 ratio). The ammonia pathway, located in the cytosol and peroxisome of hepatocytes converting the generated hydroxylamine under the consumption of NADH into ammonia (NOS2_Hz_; EC: 1.7.1.10; EntrezID: 1727):

$${NH}_{2}OH+NADH+H^{+}\to{NH}_{3}+H_{2}O+{NAD}^{+}$$

Besides ammonia, hydrazine is metabolized into nitrogen.^8^ A nitrogen metabolization pathway was established by integrating metabolite reaction steps leading from hydroxylamine to nitrogen (EC: 1.7.3.6 🡪 EC: 1.7.2.1 🡪 EC: 1.7.2.5 🡪 EC: 1.7.2.4) (<http://www.brenda-enzymes.org/>):

$$\left( {NH}_{2}OH+O_{2}\to{NO}_{2}+H_{2}O+H^{+} \right)*2$$

$$({NO}_{2}+ 2 NADH\to NO+H_{2}O+2 {NAD}^{+})*2$$

$$2 NO+ 2 NADH\to N_{2}O+H_{2}O+2 {NAD}^{+}$$

$$N_{2}O+ 2 NADH\to N_{2}+H_{2}O+2 {NAD}^{+}$$

The resulting net reaction (Hz_N2_) produces nitrogen under the consumption of oxygen and NADH:

$$2 {NH}_{2}OH+{4 O}_{2}+8 NADH\to N_{2}+6 H_{2}O+2H^{+}+8 {NAD}^{+}$$

## Comparison of step-size during combination of PBPK-GMSN models with *dMOMA*

To test, whether the chosen step-size affects the prediction of differential reaction rates in the combined PBPK-GSMN model with *dMOMA*, combined simulation for fast and slow acetylators receiving an oral dose of 300 mg isoniazid were simulated for 72 h (Supplementary Figure 1) and step-sizes of 1 and 10 minutes where used for model integration. The predicted differential reaction rate profiles were identical and resulted in the same flux perturbation (Supplementary Figure 2). The results indicate that each integration step is independent of the chosen step-size and that the previous and subsequent time points are exclusively relying on the applied xenobiotic reaction constraints.

## Once daily isoniazid 7 days isoniazid regimen

From our single dose simulation, we found that the hepatic metabolic network in slow acetylators is perturbed longer than 24 h after isoniazid intake. To further support our conclusions, that a continuous perturbation of the hepatic metabolic network during isoniazid treatment and might be a possible mechanistic explanation of the causes contributing to the increased susceptibility of slow acetylators experiencing toxic events during tuberculosis therapy, we conducted PBPK-GSMN simulations for a multiple once daily dosing schedule over 7 days for fast and slow acetylators (Supplementary Figure 4). Like in the single dose 24 h simulations, the peak fraction of significantly altered metabolic reactions is higher in fast acetylators, while the perturbations are lasting longer in slow acetylators. Here, due to the once daily administration regimen, a significant fraction (> 5 %) of the endogenous reactions in the metabolic network are consistently perturbed in slow acetylators.

## Supplementary Figures


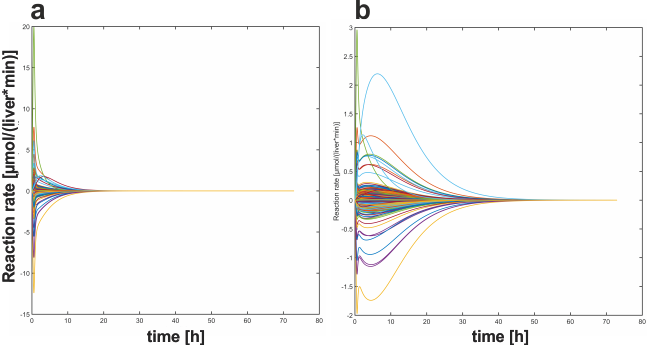


Supplementary Figure. 1: Differential reaction rate of the endogenous hepatic metabolism, simulated with combined PBPK-GSMN models after a single oral administration of 300 mg isoniazid in fast (a) and slow (b) acetylators.


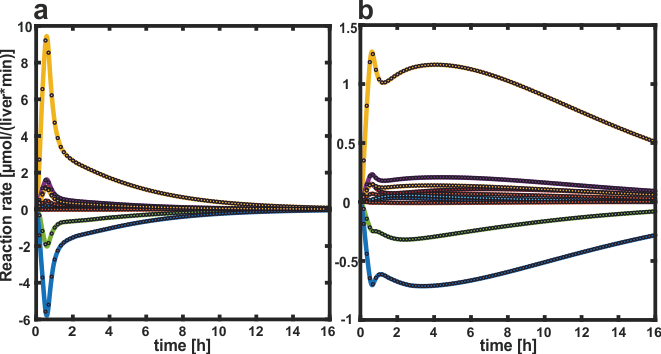


Supplementary Figure 2: Step-size comparison for a subset of simulated differential reaction rates of the combining PBPK-GSMN models and *dMOMA*. Simulation results after a single oral administration of 300 mg isoniazid in fast (a) and slow (b) acetylators. Circles represent simulations with a 10-minute step-size for *dMOMA* integration, colored lines show a 1-minute step-size.


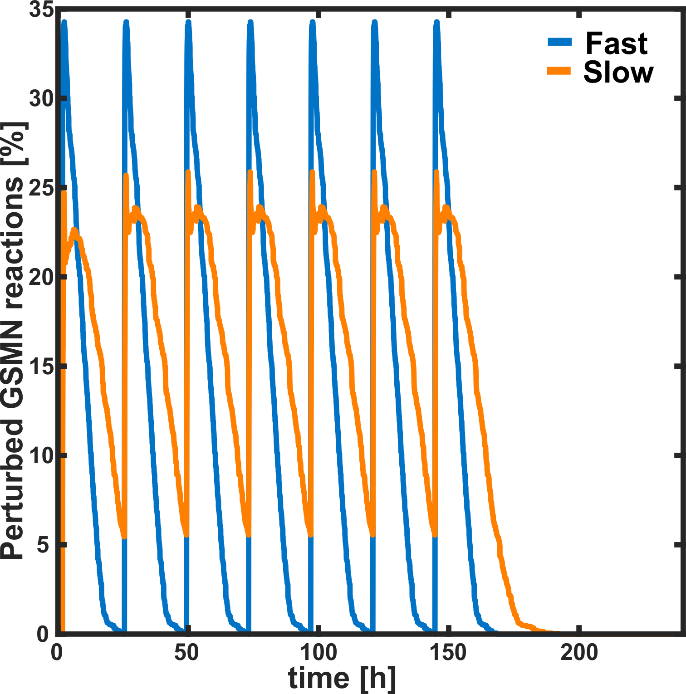


Supplementary Figure 3: Fraction of significantly altered reactions in the liver-specific GSMN models in fast (blue) and slow (orange) acetylators predicted by the combined PBPK-GSMN models after a seven consecutive oral administrations of 300 mg isoniazid.


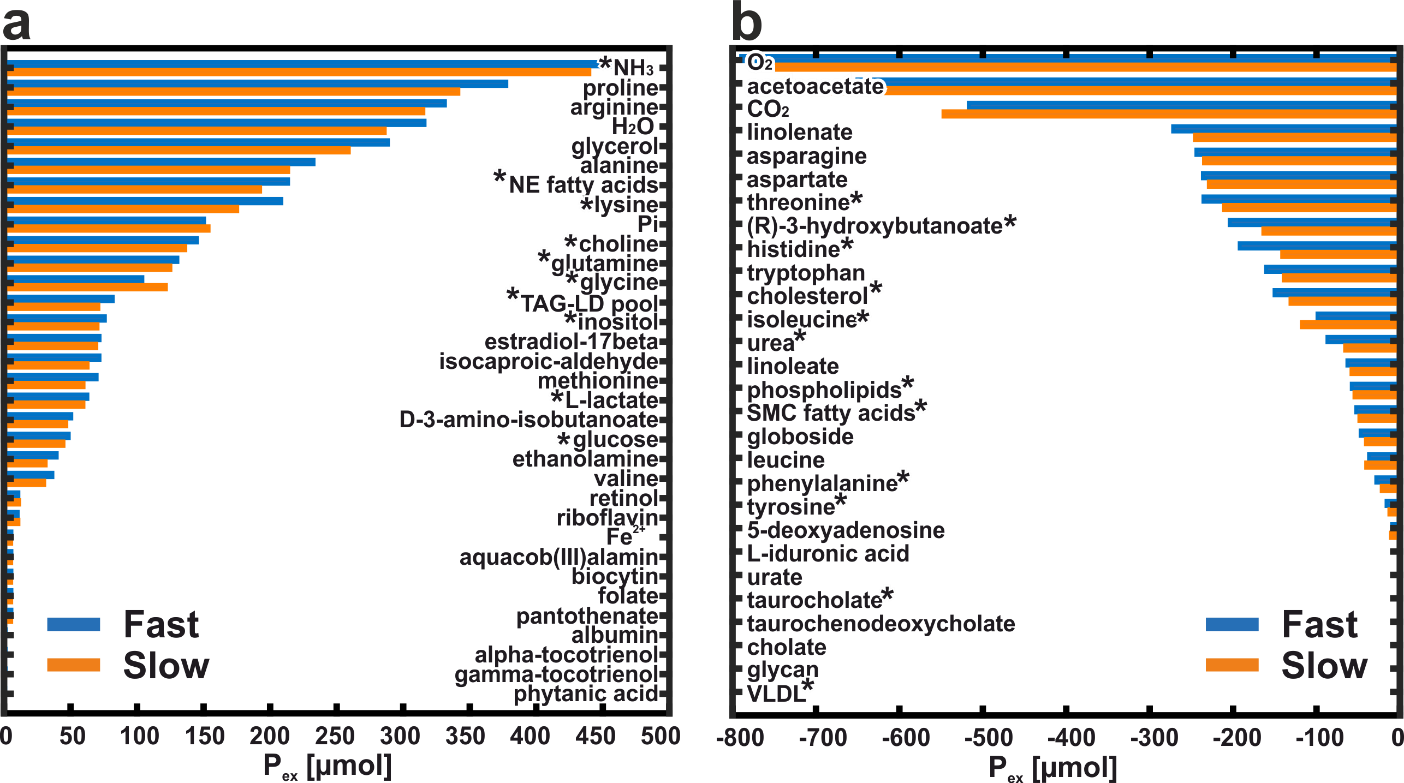


Supplementary Figure 4: Predicted changes in hepatic metabolite utilization. Increased (a) and decreased (b) hepatic exometabolome pools for fast and slow acetylators 72 h after oral administration of 300 mg isoniazid.

*Perturbed utilization in agreement with experimental and clinical literature (Supplementary Table 3)

## Supplementary References:

1. Cordes, H. *et al.* A Physiologically Based Pharmacokinetic Model of Isoniazid and Its Application in Individualizing Tuberculosis Chemotherapy. *Antimicrob. Agents Chemother.* **60,** 6134–45 (2016).

2. van der Westhuizen, F. H., Pretorius, P. J. & Erasmus, E. The utilization of alanine, glutamic acid, and serine as amino acid substrates for glycine N-acyltransferase. *J. Biochem. Mol. Toxicol.* **14,** 102–9 (2000).

3. Kubota, K. & Ishizaki, T. Dose-dependent pharmacokinetics of benzoic acid following oral administration of sodium benzoate to humans. *Eur. J. Clin. Pharmacol.* **41,** 363–368 (1991).

4. Uchino, H. Transport of Amino Acid-Related Compounds Mediated by L-Type Amino Acid Transporter 1 (LAT1): Insights Into the Mechanisms of Substrate Recognition. *Mol. Pharmacol.* **61,** 729–737 (2002).

5. Koizumi, A. *et al.* Evidence on N-acetyltransferase allele-associated metabolism of hydrazine in Japanese workers. *J. Occup. Environ. Med.* **40,** 217–22 (1998).

6. Klein, D. J. *et al.* PharmGKB summary: isoniazid pathway, pharmacokinetics. *Pharmacogenet. Genomics* **26,** 436–444 (2016).

7. Meyer, M., Schneckener, S., Ludewig, B., Kuepfer, L. & Lippert, J. Using expression data for quantification of active processes in physiologically based pharmacokinetic modeling. *Drug Metab. Dispos.* **40,** 892–901 (2012).

8. Mörike, K., Koch, M., Fritz, P., Urban, W. & Eichelbaum, M. Identification of N2 as a metabolite of acetylhydrazine in the rat. *Arch. Toxicol.* **70,** 300–5 (1996).

9. Springer, D. L., Krivak, B. M., Broderick, D. J., Reed, D. J. & Dost, F. N. Metabolic fate of hydrazine. *J. Toxicol. Environ. Health* **8,** 21–29 (1981).
